# Strong Signals of Adverse Events in Tyrosine Kinase Inhibitor Therapy for Liver Cancer Treatment

**DOI:** 10.14740/wjon2685

**Published:** 2026-01-04

**Authors:** Wen Xuan Zhou, Jun Hao Fan, Qian Wen Ni, Ming Kai Liu, Zhen Peng, Yuan Xu, Su Su Luo

**Affiliations:** a The Third Department of Hepatic Surgery, Eastern Hepatobiliary Surgery Hospital (Second Military Medical University), Naval Medical University, Shanghai 200438, China; b Proof of Concept Center, Eastern Hepatobiliary Surgery Hospital (Second Military Medical University), Naval Medical University, Shanghai 200438, China; c Department of Maternal, Child and Adolescent Health, School of Public Health, Anhui Medical University, Hefei 230032, China; d These authors contributed equally to this paper.

**Keywords:** Adverse events, Strong signals, Tyrosine kinase inhibitors, Liver cancer, Hepatocellular carcinoma, FDA Adverse Event Reporting System

## Abstract

**Background:**

This study was to identify strong adverse event (AE) signals associated with four tyrosine kinase inhibitors (TKIs) (sorafenib, regorafenib, lenvatinib, and cabozantinib), and compare these signals with regulatory drug facts from multiple global agencies.

**Methods:**

Data from the US Food and Drug Administration (FDA) Adverse Event Reporting System (FAERS, 2007 - 2024) were analyzed. Each AE was treated as a binary variable, and logistic regression with robust error estimation was used to identify strong signals (odds ratio > 2, lower 95% confidence interval > 1). AE signals were compared with drug facts from the FDA (USA), European Medicines Agency (EMA, European Union), Pharmaceuticals and Medical Devices Agency (PMDA, Japan), and National Medical Products Administration (NMPA, China).

**Results:**

Among 33,801 identifiers (137,345 records), 816 strong AE signals were found. Sorafenib had the most (373), followed by regorafenib (207), lenvatinib (126), and cabozantinib (110). Notable AEs included pharyngeal hemorrhage (sorafenib), retinal artery occlusion (regorafenib), intracranial aneurysm (lenvatinib), and mood swings (cabozantinib). Thirty-two signals had a 100% likelihood of critical outcomes, with no overlap across drugs. AEs were more frequent in males and older populations. Significant discrepancies in AE profiles were observed among regulatory agencies, with low overlap between FAERS and agency data.

**Conclusions:**

This study provides a comprehensive analysis of AE signals for four TKIs in liver cancer, identifying associations rather than causal relationships. The findings highlight significant variation in AE profiles and discrepancies between clinical trial data and real-world evidence. These results are hypothesis-generating and emphasize the need for personalized treatments, enhanced monitoring and intervention, and improved global AE reporting, while acknowledging the inherent limitations of spontaneous reporting systems.

## Introduction

Tyrosine kinase inhibitors (TKIs) have emerged as a crucial class of targeted therapies for liver cancer, particularly hepatocellular carcinoma (HCC) [1-3]. These drugs inhibit specific enzymes involved in cancer cell signaling pathways, effectively disrupting cell division, growth, and survival mechanisms. In HCC treatment, TKIs such as sorafenib, regorafenib, lenvatinib, and cabozantinib target various receptors including vascular endothelial growth factor receptors (VEGFRs), platelet-derived growth factor receptors (PDGFRs), and fibroblast growth factor receptors (FGFRs) [3]. By inhibiting these pathways, TKIs can reduce angiogenesis, induce apoptosis, and limit tumor growth [1-3].

HCC accounts for the majority of primary liver cancer cases, representing the sixth most common cancer worldwide with 865,269 new cases (4.3% of all cancers), and the third leading cause of cancer-related deaths, with 757,948 deaths (8.3% of all cancer deaths) in 2022 [4]. The prevalence is particularly high in parts of Asia and Africa, largely due to higher rates of hepatitis B and C infections [4]. Other risk factors included heavy alcohol consumption, aflatoxin exposure, metabolic dysfunction-associated steatotic liver disease (MASLD; formerly nonalcoholic fatty liver disease (NAFLD)), obesity, type 2 diabetes, and smoking [5]. Early-stage liver cancer typically shows no symptoms, leading to late diagnoses and contributing to a low 5-year survival rate [6-8].

While TKI medications have shown efficacy in treating advanced HCC, they are also associated with a range of adverse events (AEs) that can significantly impact patient care and outcomes. Studies report that 45-75% of patients experience grade ≥ 3 AEs [9]. A general algorithm for managing TKI-associated AEs suggests dose reduction or treatment interruption permanently for grade 3-4 AEs due to the risk of death, which may compromise treatment efficacy [9]. For instance, 68% of patients receiving regorafenib and 62% of those receiving cabozantinib required AE-related dose modifications, compared to 31% and 13% for placebo, respectively [10, 11]. Similarly, in a trial comparing lenvatinib with sorafenib as first-line treatments, 37-40% of patients receiving lenvatinib and 32-38% of those receiving sorafenib needed dose reductions or interruptions due to AEs [12].

The AE profiles among four TKI medications used in HCC treatment show both similarities and differences [9]. Sorafenib, the first VEGFR TKI approved in 2007, is widely used as a first-line treatment, with common side effects including diarrhea, fatigue, abdominal pain, hand-foot skin reaction (HFSR), and ascites [9, 13]. Regorafenib, used as a second-line treatment when sorafenib fails, exhibits similar side effects, including hypertension, HFSR, increased aspartate aminotransferase (AST), increased blood bilirubin, and fatigue [9, 10]. Newer anti-angiogenic TKI medications, such as lenvatinib and cabozantinib, have also demonstrated benefits in treating advanced HCC. Lenvatinib, approved as a first-line treatment in various regions including Japan, the USA, and Europe, is mainly associated with hypertension, weight loss, increased blood bilirubin, proteinuria, decreased appetite, and decreased platelet count [9, 12]. Cabozantinib, a second-line treatment, is associated with HFSR, hypertension, increased AST, diarrhea, fatigue, and asthenia [9, 11]. Despite these options, if treatments fail or are intolerable, the prognosis for HCC patients remains poor, emphasizing the importance of maintaining the recommended dose intensity for optimal efficacy [9]. Effective AE management is critical for preserving dose intensity, improving patient quality of life, and reducing treatment discontinuation [9].

However, comprehensive real-world data on the safety profiles of TKI medications in liver cancer have been limited; recent studies are beginning to address this gap. Notably, the REFINE study, the largest real-world cohort evaluating regorafenib to date, demonstrated a safety profile largely consistent with the pivotal RESORCE trial, including in third-line settings and in patients previously treated with immune checkpoint inhibitors [14]. Despite such emerging evidence, broad signal-detection analyses across multiple TKIs using global pharmacovigilance data remain scarce. While structured real-world cohorts like REFINE provide detailed clinical information for specific agents, FAERS offers complementary value through its broad scope of spontaneous reporting across diverse populations and extended time periods, though it lacks the clinical granularity of registry studies.

In addition, drug facts, primarily based on existing clinical trial data, can slightly vary across different countries and regions, such as the US Food and Drug Administration (FDA), the European Medicines Agency (EMA), the Japan Pharmaceuticals and Medical Devices Agency (PMDA), and the China National Medical Products Administration (NMPA). These drug facts may not fully reflect the patterns of AEs observed in real-world clinical practice due to the controlled environments and specific inclusion criteria. Big data-driven pharmacovigilance may help identify less common or delayed AEs that may still be clinically significant. However, comparative analysis between strong AE signals from real-world big data and those reported in drug facts remains underexplored. Therefore, the objective of this study is to investigate the strong signals of AEs associated with four TKI medications in patients living with cancer and to compare these strong signals with major drug facts from various regulatory authorities.

## Materials and Methods

### Study design and data sources

#### FAERS database

This is a retrospective, case-non-case study using the FDA Adverse Event Reporting System (FAERS) database, a public resource that collects reports of AEs, medication errors, and product quality issues related to drugs and therapeutic biological products [15]. These reports were submitted by healthcare professionals, drug manufacturers, and consumers [15]. The FAERS database has been updated quarterly since 2004 and archives individual case safety reports (ICSRs) in both ASCII and XML formats [16]. Each quarterly ASCII file includes detailed data on patient demographics (DEMO), drugs (DRUG), drug indications for use (INDI), reaction (REAC), adverse event outcomes (OUTC), sources of report (RPSR), and dates of therapy (THER) [16]. The DEMO file documents one record per event report; the DRUG and REAC files document one or more per event; the INDI, OUTC, RPSR, and THER files contain zero or more entries per event. Every file includes a unique identifier that facilitates the merging of data across different files. To achieve a large sample size and reduce year-to-year variability, we merged ASCII quarterly files from the first quarter of 2007 (2007Q1) to the latest available data, the first quarter of 2024 (2024Q1). Our analysis focused on this period following the introduction of sorafenib in 2007, the first TKI for advanced HCC treatment [17].

#### Liver cancer dataset construction

By merging the seven files (DEMO, DRUG, REAC, INDI, OUTC, RPSR, and THER), we compiled a substantial dataset with a total of 104,219,265 records. To obtain liver cancer targeted dataset, we selected records from “INDT_PT” variable in the indications for use file that included terms related to either “hepatocellular carcinoma” or “hepatic cancer”, resulting 137,345 records. For instance, records labeled “hepatocellular carcinoma non-resectable” were included because they contain the term “hepatocellular carcinoma”. Similarly, records related to hepatic cancer stages II, III, IV, metastatic, and recurrent were also included. HCC alone constituted 81.8% of our sample and hepatic cancer alone accounted for 16.2% of our sample.

To account for the influence of report volume on AE totals, we conducted subgroup analyses by randomly selecting records to match the smallest group size. For example, when comparing genders, we randomly selected 28,950 records from the male group to match 28,905 records in female group. For age comparison, since there were only 621 records for those under 18, we randomly selected 621 records from both the 18 - 65 and over 65 age groups. Similarly, when comparing the 18 - 65 group with the over 65 group, we selected 46,829 records, matching the smallest group size in this pair.

**Figure 1:**
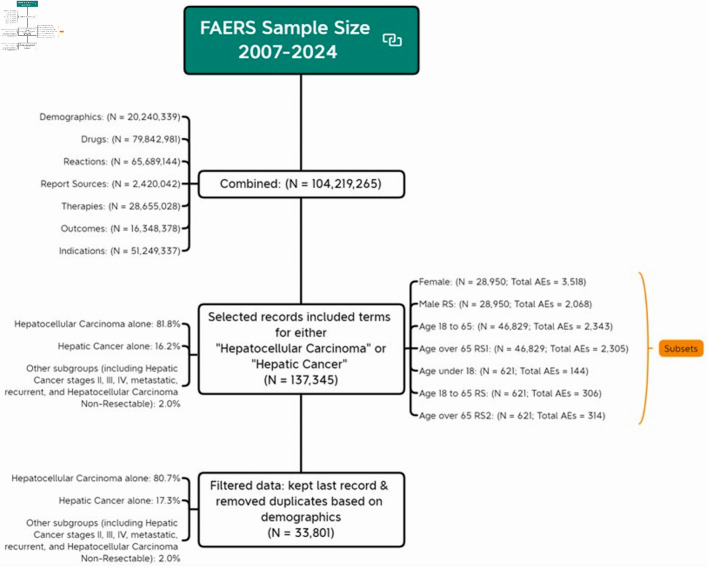
Flow chart of analytical sample screening. AE: adverse event; FDA: US Food and Drug Administration; FAERS: FDA Adverse Event Reporting System.

Demographic information was obtained by filtering the dataset to retain only the most recent record for each unique identifier, removing duplicates based on demographic factors. This resulted in 20,240,339 records. For identifiers with multiple records, we kept only the most recent one, based on the FDA received time, resulting in a total of 33,801 records. The demographic table was created using unique identifier analysis. This process is illustrated in Figure 1, which outlines the dataset screening procedure. The research protocol was approved by the Institutional Ethics Committee of Shanghai Eastern Hepatobiliary Surgery Hospital, People’s Liberation Army Naval Medical University, Shanghai, China.

### Measures

#### Primary outcome

The primary outcome was the occurrence of each AE. Since each unique identifier could be associated with one or more drug uses and zero or more AEs, we converted each AE into a binary variable, making it as present or absent for each individual. In other words, if an AE occurred for a given unique identifier, it was recorded as having that AE for that individual. The total dataset included 3,518 AEs, resulting in 3,518 binary variables. For specific subsets, the binary variables were as follows: 1,870 for females, 2,068 for randomly selected males, 144 for individuals under 18, 306 for randomly selected individuals aged 18 - 65, 314 for those over 65, 2,343 for a matched sample aged 18 - 65, and 2,305 for a matched sample over 65.

#### Primary predictor

The primary predictor of this study was TKI medications for treating liver cancer. These medicators include sorafenib, regorafenib, lenvatinib and cabozantinib. Medications were grouped based on their generic and brand names: sorafenib (Nexavar), regorafenib (Stivarga), lenvatinib (Lenvima), and cabozantinib (Cabometyx, Cometriq). All other medications included in this liver cancer treatment-focused dataset served as the control group [18]. In the overall sample, the frequencies for the sorafenib, regorafenib, lenvatinib, cabozantinib, and control groups were 25,409; 4,448; 17,576; 8,355; and 81,557, respectively.

#### Demographic and drug information

Additional demographic factors describing reported events were included. Age was recorded in units of year, month, week, day, and decade. For age grouping, months were divided by 12 to get years, weeks by 52.1775, days by 365.25, and decades were multiplied by 10 to get years. The age groups were then regrouped as < 18, 18 - 65, and > 65 years. Sex was grouped as male, female, and not specified. Outcomes were recoded into four subgroups: critical outcomes (death and life-threatening), healthcare utilization (hospitalization - initial or prolonged, and required intervention to prevent permanent impairment/damage), chronic condition (disability and congenital anomaly), and others. Occupation reporters were regrouped as medical professionals and non-medical professionals. Medical professionals included physicians, pharmacists, registered nurses, occupational therapists, and other health professionals, while non-medical professionals included lawyers and consumers. The countries of occurrence were recoded into Japan, the USA, and others, based on the proportion of AEs reported by these populations in the dataset. The types of liver cancer were regrouped into HCC alone, hepatic cancer alone, and other subgroup, which included hepatic cancer stages II, III, IV, metastatic, recurrent, and HCC non-resectable.

### Statistical analyses

#### Logistic regression for AEs

Descriptive analyses were performed to characterize the study events and calculate the percentage distribution of each drug based on unique identifiers (only the last event was selected per case ID). The full dataset, including individuals with one or more drugs and zero or more events, was used for the remainder of the analysis. Logistic regression with robust error estimation, accounting for the clustering effect of multiple AEs reported by the same individual, was used to estimate odds ratios (ORs) and 95% confidence intervals (CIs) for each AE (outcome, coded as binary variable), comparing each TKI medication to a control (predictor, coded as five categories). Each logistic regression produced an OR and 95% CI for each AE, which were then compiled. Strong signals of AEs were defined by an OR greater than 2 and a lower bound of the 95% CI greater than 1 [19], a commonly used criterion in pharmacovigilance disproportionality analysis to identify potential safety signals while acknowledging that these thresholds indicate statistical associations rather than causal relationships. These strong signals were ranked in descending order by OR, and the top 20 were selected for further reporting.

#### Strong signal identification

Strong signals indicating death and life-threatening events were identified based on percentage calculations. The percentage was determined by dividing the number of certain AE with critical condition for each drug by the total number of that AE for that drug. Strong signals with a percentage greater than 0 were considered indicative of critical conditions, with those top 20 signals being highlighted for each drug. To evaluate the similarities and differences in AEs among the four TKI medications, we summarized the counts of all possible combinations of four, three, two, and one TKI. The Jaccard similarity coefficients were calculated by determining the intersection and union of each TKI pair, with higher values indicating greater overlap. These analyses were performed across the overall dataset and seven subsets using macros. All analyses were performed using SAS version 9.4, and figures were conducted using R version 4.3.2.

### Drug facts collection and analysis

Drug facts for the four TKI medications were obtained from regulatory sources, including the FDA, EMA, PMDA, NMPA and DrugDataExpy database. These regulatory sources were selected because AEs associated with TKIs have been predominantly reported in the USA, Japan, and the European Union (EU). China was also included due to its highest number of cases and deaths associate with liver cancer [20]. As of September 5, 2024, we identified 27 approved drug facts for these four medications in liver cancer. The search terms included their generic and brand names.

AEs were extracted using the original language from the drug facts from different regulatory agencies. We then used R software to randomly select 30 AEs from multiple languages. Both Claude 3.5 and ChatGPT-4o were employed to translate these AEs into MedDRA preferred terms in English. The translation accuracy rates for Claude and ChatGPT were 90% and 80%, respectively. Additionally, a previous study reported that Claude 3.5 was the most accurate artificial intelligence (AI) in the context of astrology knowledge [21]. Therefore, we chose to use Claude 3.5 to translate AEs from different languages into MedDRA preferred terms in English. To achieve 100% accuracy, we reviewed the MedDRA preferred terms generated by Claude and double-checked them using the MedDRA software. This ensured that all AEs were standardized according to MedDRA preferred terms, maintaining consistency across regulatory data. We then compared drug facts from different regulatory agencies and identified significant signals using data from the FAERS.

The prompt we used for obtaining MedDRA preferred terms using Claude was as follow: “You are a MedDRA expert. I have some medical terms related to adverse events from clinical trials and drug facts for certain medications, and the names of these terms might not follow MedDRA rules. Could you help me convert them into MedDRA preferred terms? These terms are: (list 10 terms at a time)”

### Ethics approval

As this study was based on secondary data analysis, Institutional Review Board (IRB) approval was not required, and ethical compliance with human studies was not applicable.

## Results

### Demographic and clinical characteristics of study participants

Table 1 shows the demographic characteristics of 33,801 study records across sorafenib, regorafenib, lenvatinib, cabozantinib, and other medications. Most participants were over 65 (58.2%), followed by the 18 to 65 years group (41.3%), with a small proportion under 18 years old (0.5%). Males comprised 77.9% of the study events, females 21.8%, and 0.3% did not specify gender, consistent across all medication groups. Outcomes included 19.7% critical cases, 38.7% healthcare utilization, 0.7% chronic conditions, and 41.0% categorized as “others”. Japan (34.9%) and the USA (26.8%) were the main reporting countries. The majority of reports were from medical professionals (77.7%). HCC alone constituted 80.7% of this sample, hepatic cancer alone made up 17.3%, and other subgroups comprised 2.0%. Notable variations among medications included that cabozantinib had the highest proportion of participants over 65 (66.9%) and non-medical professionals reports (36.4%). Lenvatinib had the highest healthcare utilization (66.9%), a higher proportion of reports from Japan (68.8%), and the higher proportion of HCC cases (88.1%).

**Table 1:** Demographic Characteristics of Study Events (N = 31, 886)

Variables	Sample size	Overall	Sorafenib (n = 6,469)	Regorafenib (n = 1,294)	Lenvatinib (n = 5,862)	Cabozantinib (n = 2,440)	Others (n = 17,736)
Age							
Less than 18	133	0.5 (0.4 - 0.6)	0.6 (0.4 - 0.8)	0.1 (0.0 - 0.3)	0.1 (0.0 - 0.2)	0.1 (0.0 - 0.3)	0.7 (0.6 - 0.8)
18 to 65	10,388	41.3 (40.7 - 41.9)	50.0 (48.6 - 51.3)	51.6 (48.4 - 54.7)	34.8 (33.4 - 36.3)	33.0 (30.6 - 35.4)	40.0 (39.2 - 40.9)
Greater than 65	14,637	58.2 (57.6 - 58.8)	49.4 (48.0 - 50.8)	48.3 (45.2 - 51.5)	65.1 (63.6 - 66.6)	66.9 (64.5 - 69.3)	59.3 (58.4 - 60.1)
Gender							
Male	23,828	77.9 (77.4 - 78.4)	79.1 (78.1 - 80.1)	77.2 (74.8 - 79.6)	79.6 (78.5 - 80.6)	80.6 (79.0 - 82.2)	76.5 (75.9 - 77.2)
Female	6,665	21.8 (21.3 - 22.3)	20.6 (19.6 - 21.6)	22.8 (20.4 - 25.2)	20.4 (19.4 - 21.5)	19.4 (17.8 - 21.0)	23.0 (22.3 - 23.7)
Not specify	93	0.3 (0.2 - 0.4)	0.3 (0.2 - 0.4)	-	-	-	0.5 (0.4 - 0.6)
Outcome							
Critical outcomes	5,976	19.7 (19.2 - 20.1)	20.4 (19.4 - 21.4)	14.0 (12.1 - 15.9)	15.6 (14.7 - 16.6)	21.7 (19.3 - 24.1)	21.1 (20.4 - 21.7)
Healthcare utilization	11,757	38.7 (38.1 - 39.2)	25.0 (24.0 - 26.1)	24.0 (21.6 - 26.4)	66.9 (65.6 - 68.1)	27.5 (24.9 - 30.1)	35.9 (35.1 - 36.6)
Chronic condition	203	0.7 (0.6 - 0.8)	0.6 (0.4 - 0.8)	0.9 (0.4 - 1.4)	0.6 (0.4 - 0.8)	1.1 (0.5 - 1.7)	0.7 (0.5 - 0.8)
Others	12,465	41.0 (40.4 - 41.6)	54.0 (52.7 - 55.2)	61.1 (58.4 - 63.8)	16.9 (15.9 - 17.9)	49.7 (46.8 - 52.7)	42.4 (41.6 - 43.1)
Country of occurrence							
Japan	10,208	34.9 (34.4 - 35.5)	23.5 (22.4 - 24.6)	28.3 (25.8 - 30.8)	68.8 (67.6 - 70.1)	40.1 (38.2 - 42.1)	27.2 (26.5 - 27.9)
United States	7,825	26.8 (26.3 - 27.3)	26.1 (25.0 - 27.2)	17.7 (15.6 - 19.8)	15.8 (14.8 - 16.8)	37.8 (35.9 - 39.8)	29.9 (29.2 - 30.7)
Others	11,209	38.3 (37.8 - 38.9)	50.4 (49.1 - 51.6)	54.0 (51.3 - 56.7)	15.4 (14.4 - 16.4)	22.0 (20.4 - 23.7)	42.9 (42.1 - 43.7)
Occupation of reporter							
Medical professionals	26,097	77.7 (77.2 - 78.1)	71.6 (70.5 - 72.7)	77.3 (75.0 - 79.6)	80.9 (79.9 - 81.9)	63.6 (61.7 - 65.5)	80.8 (80.2 - 81.4)
Non-medical professionals	7,500	22.3 (21.9 - 22.8)	28.4 (27.3 - 29.5)	22.7 (20.4 - 25.0)	19.1 (18.1 - 20.1)	36.4 (34.5 - 38.3)	19.2 (18.6 - 19.8)
Type of cancer							
Hepatocellular carcinoma	27,279	80.7 (80.3 - 81.1)	77.5 (76.5 - 78.5)	83.1 (81.0 - 85.1)	88.1 (87.2 - 88.9)	82.6 (81.1 - 84.1)	79.0 (78.4 - 79.6)
Hepatic cancer	5,832	17.3 (16.9 - 17.7)	20.1 (19.1 - 21.1)	15.7 (13.7 - 17.7)	11.6 (10.7 - 12.4)	17.2 (15.7 - 18.7)	18.2 (17.6 - 18.8)
Others	690	2.0 (1.9 - 2.2)	2.4 (2.0 - 2.8)	1.2 (0.6 - 1.8)	0.4 (0.2 - 0.5)	0.2 (0.0 - 0.3)	2.8 (2.5 - 3.0)

### Strong signals of AEs across four medications

**Figure 2:**
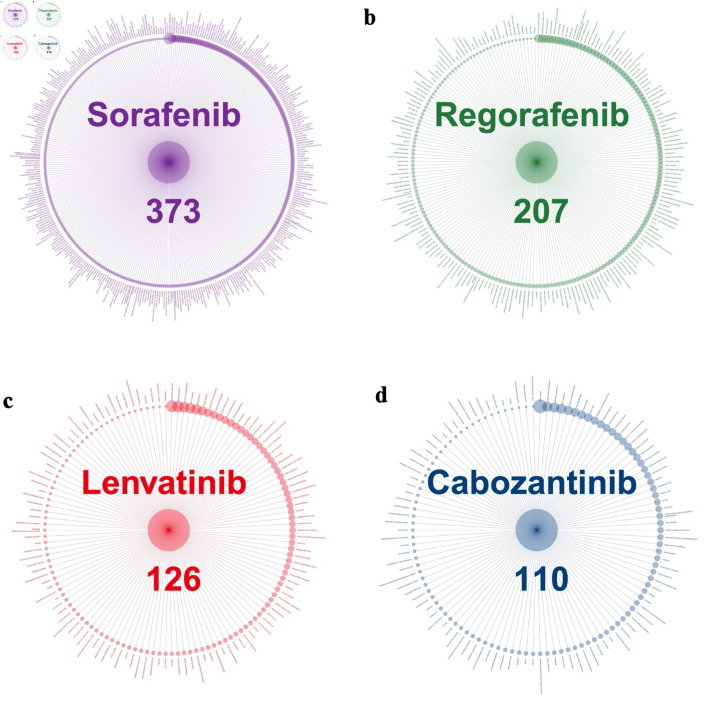
Strong signals of adverse events associated with TKI medications. (a) Sorafenib. (b) Regorafenib. (c) Lenvatinib. (d) Cabozantinib. TKI: tyrosine kinase inhibitor.

Figure 2a-d presents the distribution of strong signals of AEs associated with the four TKIs. Sorafenib had the strongest signals (373), followed by regorafenib (207), lenvatinib (126), and cabozantinib (110). The size of dots represents the magnitude of the OR with larger dots indicating higher OR values. The nature and the OR of AEs varied significantly across medications. The full list of strong signals, including their names and ORs, is provided her (Supplementary Material 1, wjon.elmerpub.com).

**Figure 3:**
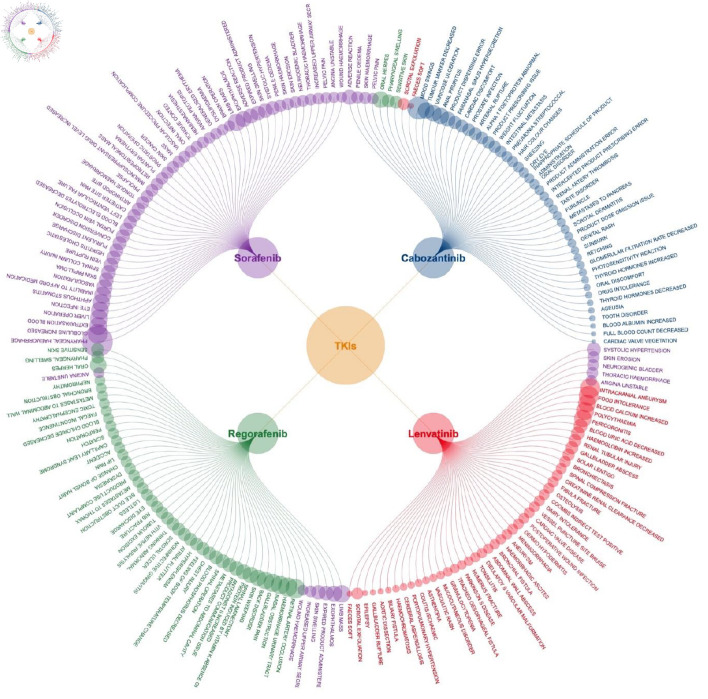
Top 50 strong signals of adverse events associated with four TKI medications. TKI: tyrosine kinase inhibitor.

To highlight key strong signals, Figure 3 presents the top 50 strong signals for each medication, and the top three for each drug are noted here. For sorafenib, these included pharyngeal hemorrhage (OR = 204.8), increased globulins (OR = 74.2), and extravasation blood (OR = 57.9); for regorafenib, retinal artery occlusion (OR = 73.5), hemorrhage urinary tract (OR = 69.0), and nasal obstruction (OR = 63.6); for lenvatinib, intracranial aneurysm (OR = 76.7), food intolerance (OR = 74.3), and increased blood calcium (OR = 58.1); for cabozantinib, mood swings (OR = 104.5), decreased tumor marker (OR = 48.9), and varicose ulceration (OR = 48.8).

**Figure 4:**
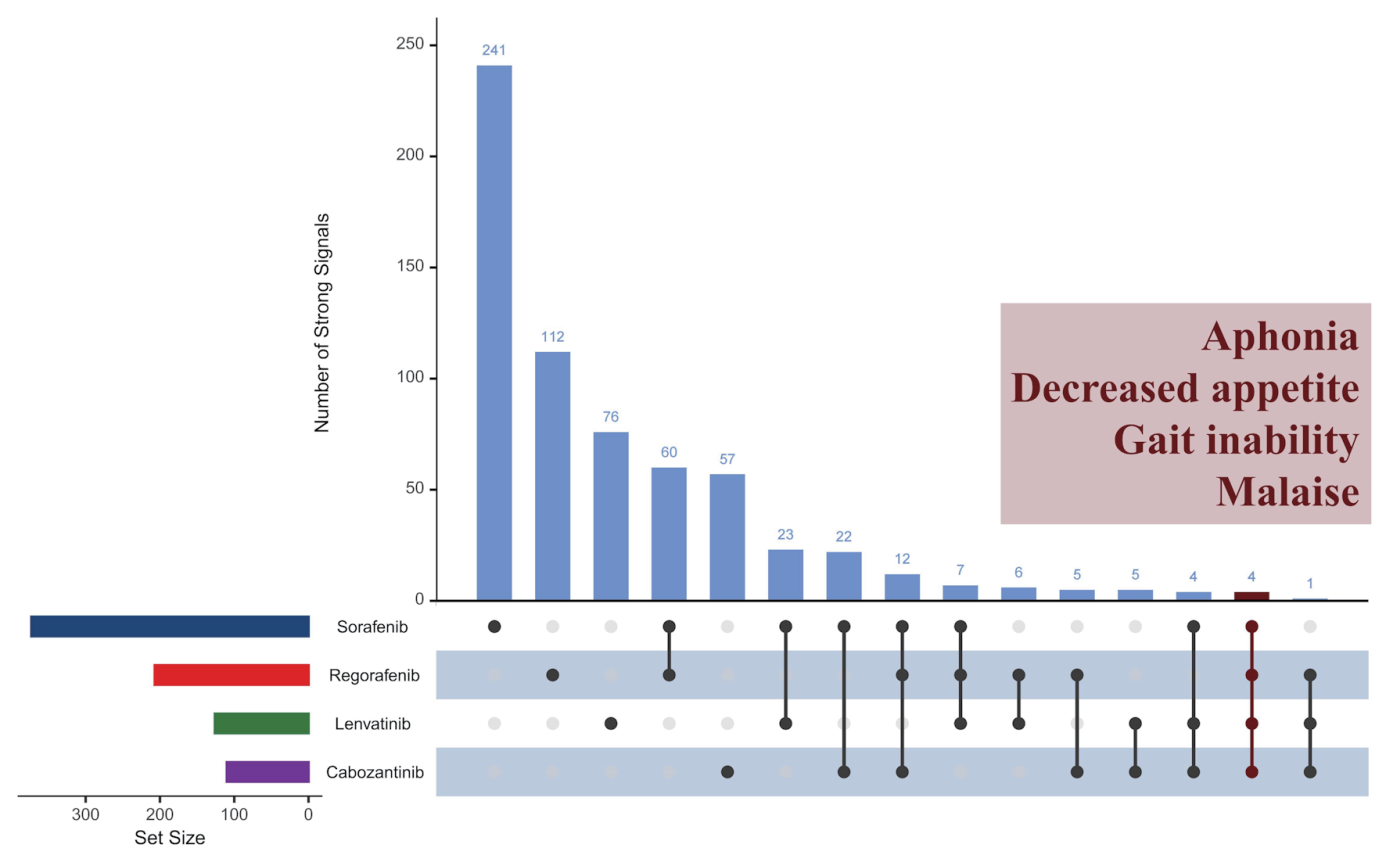
Comparison of strong signals of adverse events across four TKI medications in overall population. TKI: tyrosine kinase inhibitor.

Figure 4 shows similarities and the differences across four TKI medications. In the overall population, 816 strong signals were identified (635 unique and 149 shared). This indicates that 19.0% (149/(635 + 149)) of the strong signals occurred in more than one drug. Only four strong signals - aphonia, decreased appetite, gait inability, and malaise - were shared by all four drugs. Among three medications, sorafenib, regorafenib, and cabozantinib shared 12 signals, while sorafenib, regorafenib, and lenvatinib shared seven signals. Sorafenib, lenvatinib, and cabozantinib shared four signals, and regorafenib, lenvatinib, and cabozantinib shared one. Regarding paired medications, sorafenib shared the most AEs with regorafenib (60 signals), followed by lenvatinib (23) and cabozantinib (22). Each drug alone accounted for the majority of its AEs: sorafenib, regorafenib, lenvatinib, and cabozantinib individually accounted for 64.6%, 54.1%, 60.0%, and 51.8% of their signals, respectively. The Jaccard similarity index analysis for each drug pair further confirmed these findings, with indices ranging from 0.06 to 0.17, indicating low overlap in AEs between paired drugs.

### Strong signals among subgroups

**Figure 5:**
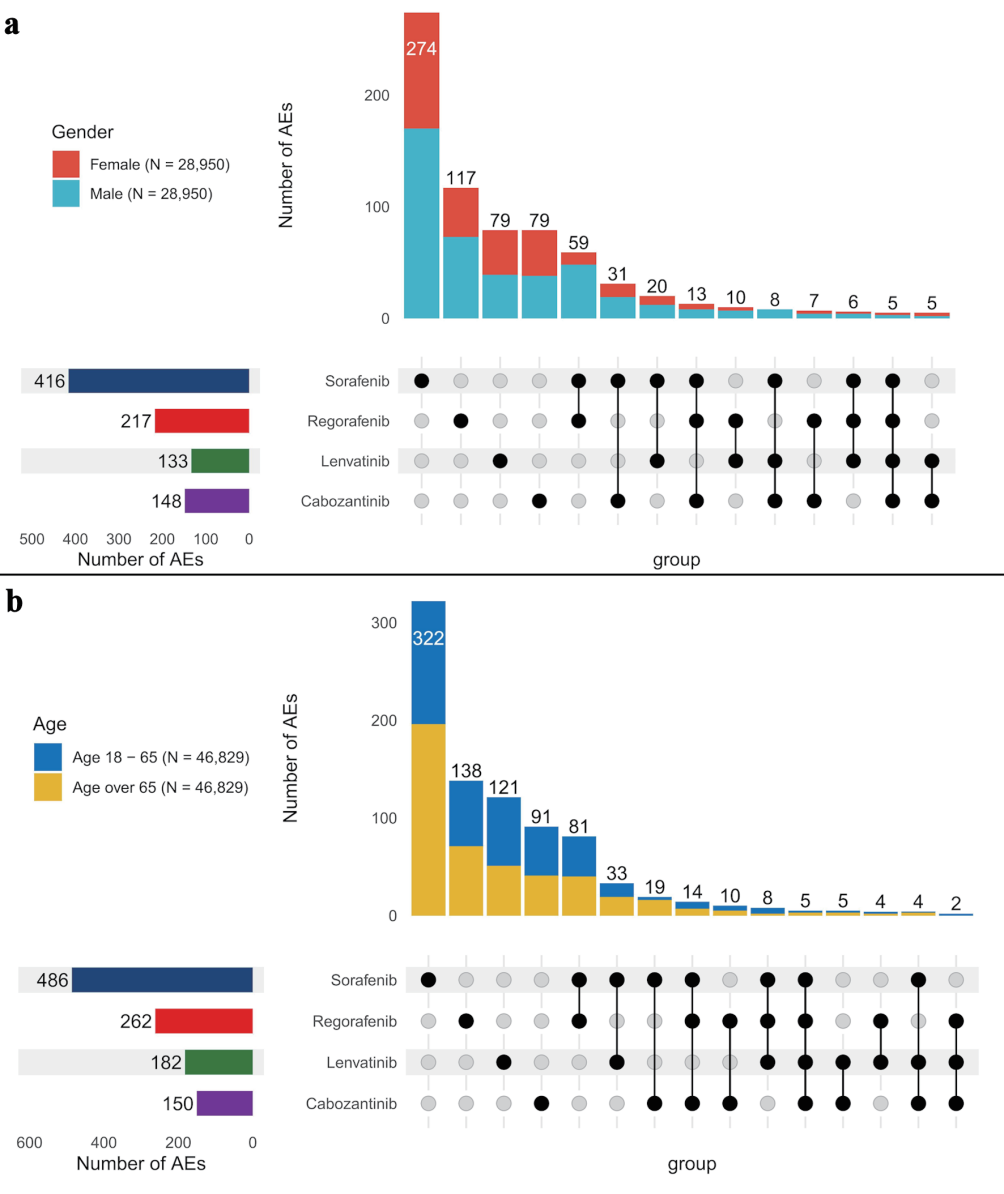
Comparison of strong signals of adverse events across four TKI medications by gender (a) and age groups (b). AE: adverse event.

Strong signals varied across gender (Fig. 5a) and age subgroups (Fig. 5b). Females exhibited 338 strong signals, while a randomly selected male subgroup showed a higher count of 576 signals. To ensure a balanced comparison, the male sample was matched to the female sample size, and the same strategy was applied to age comparisons. In terms of age, the older age group seemed to report a higher number of signals. Individuals aged 18 - 65 (49 signals) and over 65 (48 signals) reported more signals than those under 18 (37 signals). Although the 18 - 65 and over-65 groups reported similar numbers of signals in a smaller sample (n = 621), the over-65 group had significantly more signals (577) compared to the 18 - 65 group (503) in a larger sample (n = 46,829). This study primarily focused on reporting AEs in the overall population, with subgroup analysis offering insights into group differences, though the full list of AEs was not included.

**Figure 6:**
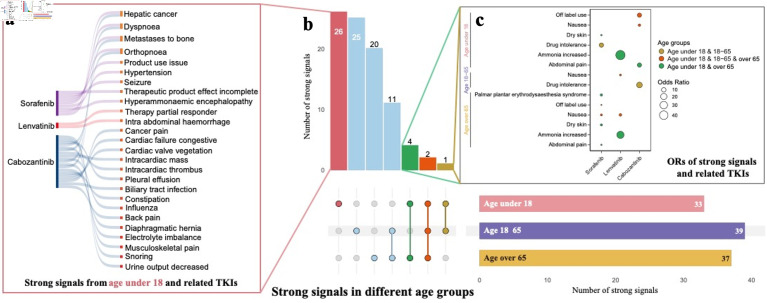
(a, b, c) Comparison of strong signals of adverse events across four TKI medications between age under 18, age 18 - 65, and age over 65. TKI: tyrosine kinase inhibitor.

To specifically investigate strong signals in individuals under 18, we presented the strong signals identified across the four medications for the age groups under 18, 18 - 65, and over 65 (Fig. 6a-c). For sorafenib, palmar-plantar erythrodysesthesia syndrome was reported in all age groups. However, 11 additional strong signals, such as food intolerance, respiratory issues, seizures, bone displacement, and high oxygen saturation, were exclusive to participants under 18. Regorafenib showed no AE signals in the under 18 group, while some AEs were reported in adults and seniors. Lenvatinib was associated with appetite loss across all age groups, with three additional AEs - high blood calcium levels, therapy area involvement, and abdominal hemorrhage - reported only in the under 18 group. Adults and seniors shared fatigue and asthenia as common AEs. Similarly, cabozantinib also showed appetite loss across all age groups, with 20 additional AEs reported only in participants under 18, including pain, bone displacement, heart issues, and fluid accumulation. These findings highlight distinct AE patterns across age groups for each TKI.

### Strong signals indicating critical conditions

Among 816 strong signals overall, 540 signals indicated critical conditions. Sorafenib had the most (244), followed by lenvatinib (131), regorafenib (90), and cabozantinib (75). The full list of critical signals for each medication is provided here (Supplementary Material 2, wjon.elmerpub.com). These signals were more frequently observed in males and older age groups, reinforcing the trends seen in the overall strong signals. Notably, 32 signals were associated with a 100% rate of developing critical outcomes: 11 for sorafenib, seven for regorafenib, 10 for lenvatinib, and four for cabozantinib. For sorafenib, examples include bacterial diarrhea and hemorrhagic ascites; for regorafenib, ischemic colitis and bladder disorder; for lenvatinib, cachexia and urosepsis; and for cabozantinib, arterial rupture and cholelithiasis. No overlapping signals were found across the four drugs.

### Drug facts comparison

Table 2 compares the strong signals from FAERS with the adverse reactions (ARs) approved by regulatory agencies, including the FDA (USA), EMA (EU), PMDA (Japan), and NMPA (China). For sorafenib, the FDA, EU, Japan, and China reported 129, 110, 117, and 107 ARs, respectively, but only 34, 24, 25, and 25 were consistent with FAERS. For regorafenib, the agencies reported 43, 65, 129, and 61 ARs, with consistent signals of eight, nine, 16, and eight. Lenvatinib exhibited greater variability, with Japan and China reporting 129 and 167 ARs compared to the FDA (105) and EU (88), though consistent signals were low (16 and 14). For cabozantinib, the FDA and EU reported 114 and 166 ARs, with 17 and 16 consistent signals, respectively. Variability in inter-agency comparisons ranged from 33 for lenvatinib between the FDA and China to 95 for sorafenib between the EU and China.

**Table 2:** Comparison of Strong Signals From FAERS and ARs Reported by Four Regulatory Agencies

	Sorafenib	Regorafenib	Lenvatinib	Cabozantinib
FAERS	373	207	126	110
Drug facts				
FDA	129	43	105	114
EU	110	65	88	166
Japan	117	129	87	73
China	107	61	167	-
Consistent AEs				
FDA	34	8	11	17
EU	24	9	8	16
Japan	25	16	7	11
China	25	8	14	-
Comparison between agencies				
FDA vs. EU	90	35	57	73
FDA vs. Japan	88	38	48	51
FDA vs. China	87	33	83	-
EU vs. Japan	92	58	49	60
EU vs. China	95	59	82	-
Japan vs. China	93	53	60	-

AE: adverse event; AR: adverse reactions; FDA: US Food and Drug Administration; FAERS: FDA Adverse Event Reporting System; EU: European Union.

## Discussion

### Interpretation of findings

This study provides a comprehensive analysis of AEs associated with TKI medications in liver cancer treatment using FAERS data from 2007 to 2024. Overall, we identified 816 strong signals of AEs, with 540 of these signals indicating death or life-threatening condition across four TKI medications. The results highlight substantial risks, with significant variation in the nature and severity of AEs among these TKI medications across the overall population and subgroups. In addition, this study reported discrepancies between our strong AE signals and those listed in the drug facts across four regulatory agencies. To our knowledge, this is the first thorough assessment of the AE profiles of these four major TKI medications in liver cancer treatment based on a large real-world dataset.

### Comparison with previous studies for strong signals

Sorafenib, as the first TKI approved for HCC [17], exhibited the highest number of strong signals (373), followed by regorafenib with 207 signals, while lenvatinib and cabozantinib had relatively fewer signals, with 126 and 110, respectively. These large numbers of strong signals identified indicate a broad range of AEs. However, it is important to note that the higher number of strong signals for sorafenib does not necessarily indicate a higher occurrence rate of AEs compared to other TKIs [15]. The number of AEs identified might be influenced by the total number of records reported for each drug, as higher usage provides more opportunities to observe and document these events. In our study, reports indicated that sorafenib was used by 6,469 unique identifiers, followed by lenvatinib (5,862), cabozantinib (2,440), and regorafenib (1,294). Consequently, the rate of strong signals per unique identifier was 5.8% for sorafenib, 16.0% for regorafenib, 2.2% for lenvatinib, and 4.5% for cabozantinib. To our knowledge, the number of strong signals and these rates by TKI have not been previously reported, providing valuable information for risk assessment and informed decision-making in TKI therapy.

Moreover, while a big number of 816 signals were identified for the four TKI medications, the overlap among them was limited, with only about 20% of signals appearing in two or more drugs. We also identified 32 signals that indicate a 100% likelihood of death or life-threatening outcomes, but no identical signals were found across all four drugs. This finding aligns with previous studies that reported frequent occurrences of severe AEs in patients treated with TKIs [9-13]. However, our results find that the specific AEs associated with different TKIs vary considerably, which contrasts with earlier research. Previous studies had reported that the most common AEs including fatigue, diarrhea, HFSR, nausea, vomiting, decreased appetite, hypertension, and weight loss, were generally similar across the four TKI medications [9]. Other studies have also consistently reported these common AEs in TKI medications for liver cancer treatment [22-26]. One possible reason is that previous clinical trials often focused on relatively short-term effects, while our study includes data starting from the first TKI on the market, capturing potential long-term effects from years of TKI use. For example, Schwartz et al reported that in treating HCC with cabozantinib, the median time to the first occurrence of an AE ranged from 0 to 16 weeks, with hypertension occurring as early as 2 weeks and fistulas as late as 14 weeks [22]. As a result, similar AEs reported across different TKI medications might suggest similar strategies for managing side effects.

Actually, the diverse strong signals identified for each drug reveal distinct AE profiles among these TKIs. These results emphasize the need for individualized patient management when prescribing these medications. Common grade ≥ 3 AEs and the strong signals can complement each other in assessing and managing the risks associated with drug therapies. Future studies may further examine potential causal relationships among the top signals, particularly those linked to critical outcomes or shared across multiple medications, to deepen understanding of their clinical relevance. Given that nearly half of the patients experience AEs leading to dose interruptions or reductions, which ultimately impact survival and treatment efficacy [9-11, 13, 22, 27], robust patient education programs about potential risks and patients care are essential. Healthcare providers should carefully weigh the benefits against their potential risks, managing these risks through regular follow-ups and supportive care. Future research should focus on balancing efficacy and safety in TKI treatments; an emphasis on developing targeted interventions is crucial to reduce harm and to improve patient outcomes as well as quality of life.

### Subgroup analysis and vulnerable populations

The subgroup analyses highlight increased vulnerability in males and older adults, who report more strong signals than their counterparts. Regarding age, our findings align with previous research, suggesting that older adults may be more susceptible to the side effects of cancer treatments, likely due to a combination of comorbidities, altered pharmacokinetics, and the physiological effects of aging [28, 29]. In terms of gender difference, Unger et al who reviewed data from SWOG phase II and III clinical trials (1980 - 2019), involving 23,296 patients and 274,688 AEs, found that women had significantly higher risks of severe AEs: 25% higher for targeted therapy, 36% for chemotherapy, and 49% for immunotherapy [30]. This discrepancy may stem from differences in AE reporting, pharmacogenomics, dosage, and adherence [30]. However, our study found more AEs reporting in males than females. To be honest, we do not have a good explanation for now. Further research is needed to consider the difference in AEs between genders.

### Discrepancies in regulatory reporting

In terms of drug facts, we found considerable variation in AEs reporting across the four regulatory agencies for the same drug. This aligns with previous findings from other drug studies [31]. Additionally, our analysis found low consistency in AEs reporting between FAERS and the four regulatory agencies. For example, although sorafenib had a relatively higher similar AEs reported across the FDA, EU, Japan, and China, the number of consistent signals with FAERS was low, indicating that the methodologies used by each agency to monitor and report ARs vary considerably. This pattern was also observed with other drugs. These inconsistencies may stem from factors such as differences in strong signals detection algorithms, causal relationship establishment, reporting frequency, and update timelines between FAERS and regulatory sources. It might be valuable to explore methods to enhance strong signal detection across systems.

### Clinical implications

The strong AE signals identified in this study, while hypothesis-generating rather than confirmatory, offer several clinical implications. Notable signals absent from current drug labels, including pharyngeal hemorrhage for sorafenib, retinal artery occlusion for regorafenib, intracranial aneurysm for lenvatinib, and mood swings for cabozantinib, may warrant enhanced clinical vigilance during routine monitoring. The limited 20% overlap in signals across TKIs suggests that each agent requires individualized safety surveillance protocols rather than a uniform approach.

In addition, the low concordance between FAERS strong signals and regulatory drug-label information likely reflects several structural differences between data sources. FAERS captures spontaneous, real-world reports from diverse clinical settings, whereas regulatory drug facts rely heavily on controlled clinical trial data with selective eligibility criteria, standardized follow-up, and predefined reporting protocols. Variations in regional reporting requirements, differences in strong-signal detection methodologies, and the timing and frequency of post-marketing updates may also contribute to these discrepancies.

Such inconsistencies may influence clinical practice by highlighting AE patterns that clinicians may encounter in routine care but that are not yet represented in drug labeling. Therefore, integrating pharmacovigilance data with existing clinical evidence may help clinicians remain alert to emerging risks, refine patient counseling, and develop more comprehensive, liver-function-aware risk-stratification and monitoring strategies tailored to individual patients and specific TKI agents.

### Limitations and strengths

The study also has several limitations. First, as with all spontaneous reporting systems, the FAERS database is subject to inherent biases, including underreporting, heterogeneity in reporting quality, duplicate submissions, and potential inaccuracies in symptoms reported by patients or consumers. These issues limit the completeness and reliability of AE ascertainment. Second, FAERS lacks key clinical variables - particularly liver function parameters such as bilirubin, albumin, international normalized ratio (INR), ascites, and encephalopathy - preventing calculation of Child-Pugh or albumin-bilirubin (ALBI) scores. As a result, it is not possible to assess how residual hepatic reserve influences AE risk or to distinguish safety profiles between Child-Pugh class A and B patients, an important consideration in TKI therapy. Third, because FAERS does not capture treatment line, sequencing information, or performance status, the impact of first-line failure or deteriorating hepatic function on AE patterns could not be assessed. Fourth, nearly 70% of reports lacked complete information on drug administration dates and AE onset times, preventing time-to-event analyses that are essential for understanding temporal relationships between exposure and adverse outcomes. Fifth, due to the absence of reliable data on comorbidities, disease severity, and concomitant therapies, confounding factors could not be fully adjusted for, and the associations identified should not be interpreted as causal. Sixth, the analytic choice of using all non-TKI medications as the reference group may dilute drug-specific effects and introduce heterogeneous confounding, although it allowed the inclusion of the maximal available sample. Additionally, substantial variation in sample size across TKI groups may affect the stability and comparability of signal detection. Seventh, subgroup analyses, particularly those involving the under-18 population and gender-matched samples, are exploratory and limited by small or uneven sample sizes, making these findings less stable and requiring cautious interpretation.

Despite these limitations, the study has several notable strengths. It leverages the FAERS database from 2007 to 2024, allowing the largest and longest real-world pharmacovigilance evaluation of four major TKIs since their market introduction. By converting each AE into a binary variable for every unique identifier, the study incorporates the full spectrum of reported events rather than relying on a single selected record, thereby providing a comprehensive assessment of the AE landscape. To our knowledge, this is the first study to systematically identify, quantify, and compare strong AE signals and critical (death or life-threatening) events for sorafenib, regorafenib, lenvatinib, and cabozantinib using a unified analytical framework. The study further contributes novel insights by comparing FAERS-based strong signals with drug facts across multiple global regulatory agencies, highlighting discrepancies that underscore the complementary value of real-world pharmacovigilance data.

### Conclusions

This study identified 816 strong signals of AEs across four TKIs, with 32 signals linked to a 100% likelihood of such outcomes. Sorafenib had the highest number of strong signals (373), followed by regorafenib (207), lenvatinib (126), and cabozantinib (110). AEs varied significantly and were more commonly reported in males and older populations among the four TKI medications. In addition, significant differences in AE profiles were observed across the FDA, EU, Japan, and China. These findings highlight the need for careful patient selection, liver-function-aware treatment strategies, and proactive AE monitoring when prescribing TKIs for liver cancer. While FAERS-based signals are not causal, they can help clinicians remain alert to potential risks not yet reflected in clinical trials or regulatory labeling. Enhanced post-marketing surveillance and real-world clinical cohorts with detailed liver-function metrics are essential to clarify the causal pathways linking TKIs to adverse outcomes.

## Supplementary Material

Suppl 1 Full list of strong signals for four TKI medications.

Suppl 2 Full list of strong signals indicating critical outcomes for four TKI medications.

## Data Availability

The datasets analyzed during the current study are publicly available and were obtained from the FDA Adverse Event Reporting System (FAERS). These datasets can be accessed through the following link: https://fis.fda.gov/extensions/FPD-QDE-FAERS/FPD-QDE-FAERS.html. Analytic code used to conduct the analyses may be available by emailing the corresponding author.
